# C9orf72-ALS mutation drives basal mitophagy impairments in iNeurons

**DOI:** 10.3389/fncel.2026.1731669

**Published:** 2026-02-11

**Authors:** James A. K. Lee, Chloe Moutin, Sarah Granger, Katie Roome, Allan Shaw, Scott P. Allen, Laura Ferraiuolo, Pamela J. Shaw, Heather Mortiboys

**Affiliations:** 1 Division of Neuroscience, School of Medicine and Population and Health, Sheffield Institute for Translational Neuroscience, University of Sheffield, Sheffield, United Kingdom; 2 NIHR Sheffield Biomedical Research Centre, Sheffield Teaching Hospitals NHS Foundation Trust, Sheffield, United Kingdom; 3 Neuroscience Institute, University of Sheffield, Sheffield, United Kingdom

**Keywords:** ALS (Amyotrophic lateral sclerosis), autophagy, mitochondria, mitophagy, ULK1

## Abstract

**Introduction:**

ALS is a neurodegenerative disorder characterized by progressive upper and lower motor neuron loss. A GGGGCC hexanucleotide repeat expansion (HRE) in the C9orf72 gene is the most common mutation found in populations of European descent. Mitochondrial dysfunction has been observed in C9orf72-ALS patients and models of the disease, however, reports on mitochondrial clearance via mitophagy in C9orf72-ALS are limited.

**Results:**

iNeurons from C9orf72-ALS patients displayed reduced mitochondrial membrane potential and reduced basal mitophagy, due to reductions in autophagosome production and reduced ULK1 recruitment to mitochondria. No consistent changes to PINK1/Parkin or BNIP3 mitophagy pathways were observed.

**Conclusion:**

Our data show that certain aspects of mitochondrial function is impaired in C9orf72-ALS patient iNeurons. An in-depth characterization of mitophagy suggests that a deficit in autophagosome production is responsible and provides further evidence that toxic gain-of-function mechanisms in C9orf72-ALS are responsible for autophagy deficits.

## Introduction

Amyotrophic lateral sclerosis (ALS) is the most common subtype of motor neuron disease, characterized by progressive loss of upper and lower motor neurons. Resulting symptoms include progressively developing muscle weakness and paralysis. In cohorts of European descent, the most commonly identified genetic subtype is a hexanucleotide repeat expansion in the first intron of C9orf72 (Woollacott and Mead, 2014). Multiple pathogenic mechanisms have been identified to contribute to neuronal toxicity in C9orf72-ALS, including haploinsufficiency, sequestration of RNA-binding proteins at RNA foci, and generation of dipeptide repeat proteins (DPR’s) from RAN translation of the repeat expansion (DeJesus-Hernandez et al., 2011; Renton et al., 2011) These mechanisms appear to synergise to contribute to neurodegeneration, with RNA foci and DPR production thought to be the primary drivers of neuron loss and reductions in the C9orf72 protein exacerbating the pathophysiology (Braems et al., 2020).

There is a wealth of evidence describing mitochondrial dysfunction in ALS models [reviewed recently by Lee et al. (2024)]. Dysfunctional mitochondria have also been observed in C9orf72-ALS models, with a majority of reports suggesting increased mitochondrial fission and disrupted mitochondrial function (Choi et al., 2019; Dafinca et al., 2016; Li et al., 2020; Lopez-Gonzalez et al., 2016; Mehta et al., 2021; Onesto et al., 2016; Petrozziello et al., 2022). We have also previously shown increases in mitochondrial reactive oxygen species (ROS) production in C9orf72-ALS iNeurons, a finding corroborated in *Drosophila* models of C9orf72-ALS (Au et al., 2024). These dysfunctional mitochondria may accumulate in cells due to impaired clearance via mitophagy. Reductions in mitophagy have been reported in *Drosophila* expressing arginine-rich DPR’s and in zebrafish models combining C9orf72 loss and polyGP expression, however, these findings have not yet been confirmed in patient-derived neuronal models (Au et al., 2024; de Calbiac et al., 2024).

The present study utilizes neuronal progenitor cells directly reprogrammed from patient fibroblasts, allowing us to retain the genetic background and age phenotype of donor cells (Gatto et al., 2021; Meyer et al., 2014). We have previously shown that generation of iNeurons from iNPC’s results in cells expressing neuronal markers β-III tubulin, MAP2 and NeuN (Au et al., 2024). Furthermore, we have previously shown these C9orf72 patient derived cells express polyGP dipeptide repeats, providing further validation that this model is relevant for the study of C9orf72 related mechanisms (Hautbergue et al., 2017; Castelli et al., 2023). The present study includes an extensive characterization of mitochondria and mitophagy in a patient-derived model of C9orf72-ALS, building on previous work showing disruptions to mitophagy in neurons of other ALS genotypes. We show iNeurons carrying the C9orf72-HRE mutation are deficient in basal mitophagy, with reductions in autophagosome generation. In contrast to other ALS genotypes, no defects in PINK1/Parkin-dependent and no consistent changes in BNIP3/BNIP3L-dependent mitophagy were observed. Modulation of ULK1 was able to elicit an increase in mitophagy, however, levels were still reduced overall in the C9orf72-ALS patient iNeurons. This study provides evidence that disruption to the autophagy machinery produces deficits in mitophagy in C9orf72-ALS, with evidence that toxic gain-of-function mechanisms are responsible for autophagy disruption.

## Materials and methods

### iNPC tissue culture, iNeuron differentiation, and compound treatments

Induced Neuronal Progenitor Cells (iNPC’s) were generated as previously described (Meyer et al., 2014). Table 1 includes details for the patient lines used in this study. The generation and use of cells derived from these iNPC’s has been published before in Meyer et al. (2014) and Au et al. (2024). iNPC’s were maintained in DMEM: F12 with GlutaMAX (Gibco), supplemented with 1% N2 (Invitrogen), 1% B27 (Invitrogen) and 20 ng/ml FGF-Basic (PeproTech). Cells were grown on fibronectin (R&D Biosystems) coated cell culture dishes and routinely sub-cultured every 2–3 days using Accutase (Corning) to detach them.

**TABLE 1:** Cell lines used in this study.

Cell line	Genotype	Sex	Age at biopsy (years)	Source	Onset to death (months)
155v2(pair1)	Control	Male	40	Sheffield teaching hospital (STH)	-
3,050 (pair 2)	Control	Male	68	STH	-
CS14 (pair3)	Control	Female	52	Cedars-Sinai	-
183 (Pair 1)	C9orf72-ALS	Male	50	STH	27
78 (Pair 2)	C9orf72-ALS	Male	66	STH	31.7
ALS52 (pair 3)	C9orf72-ALS	Male	49	Cedars-Sinai	57

To achieve neuronal differentiation, iNPC’s were plated into six well plates and allowed to reach ∼80% confluency. iNeuron differentiation was started by switching to DMEM: F12 media with GlutaMAX supplemented with 1% N2, 2% B27 and 2.5 μM DAPT (Sigma). After 48 h, DAPT was removed and replaced in media with 1 μM retinoic acid (Sigma), 1 μM smoothened agonist (Millipore) and 2.5 μM Forskolin (Cayman Chemical). Cells were maintained in this media for 16 days before being used in assays.

To assess dependency of neurons on OXPHOS or glycolysis for ATP production throughout differentiation, cells were treated with 10 μM oligomycin to inhibit OXPHOS, 50 mM 2-deoxyglucose to inhibit glycolysis, or both simultaneously for 30 min at 37°C, before ATP measurements were performed with the ATPlite Luminescence Assay as per the manufacturers instructions (Revvity). From this we can calculate the percentage of ATP levels, setting untreated at each time point to 100%.

For deferiprone treatments, cells were treated with either 500 μM or 1 mM for 24 h prior to fixing cells and immunostaining. For oligomycin/antimycin A treatments, cells were treated with 10 μM oligomycin and 4 μM antimycin A for 1 h before fixing cells and immunostaining. For nilotinib, BL-918 and A769662 treatments, cells were treated with 1 or 5 μM nilotinib/BL918, or 1 or 10 μM A769662, for 24 h prior to fixing cells and immunostaining. For autophagy induction, cells were treated with either 200 nM bafilomycin A1, 1 μM rapamycin, 15 μM chloroquine or 1μM rapamycin/15 μM chloroquine in combination for 5 h before fixing cells and immunostaining.

### Live imaging of iNeurons

For mitochondrial membrane potential (MMP) measurements, cells were stained for 1 h with 20 μM Hoechst, 80 nM TMRM and 200 nM MitoTracker Green diluted in MEM. Cells were imaged using an Opera Phenix high content imaging system (Revvity) with a 40x water immersion objective across 15 fields of view and 6 Z stacks. Cells were maintained at 37°C and 5% CO_2_ throughout imaging. Image analysis was performed using a custom protocol in Harmony 4.9 (Revvity).

For live mitophagy flux measurements, cells were stained for 1 h with 20 μM Hoechst, 80 nM tetramethylrhodamine methyl ester (TMRM), 50 nM Lysotracker DeepRed and 200 nM MitoTracker Green diluted in MEM. For induced cells, 10 μM oligomycin and 4 μM Antimycin A were added immediately before imaging. Cells were imaged using an Opera Phenix high content imaging system (Revvity) with a 40x water immersion objective across 9 fields of view and 4 Z stacks, with imaging repeated every 30 min for 90 min. Cells were maintained at 37°C and 5% CO_2_ throughout imaging. Image analysis was performed using a custom protocol in Harmony 4.9 (Revvity).

### Respirometry measurements

iNeurons were plated at 20,000 cells per well in a fibronectin-coated 96-well Seahorse cell culture plate (Agilent) in iNeuron media and incubated overnight at 37°C and 5% CO_2_. The following day, media was replaced with Seahorse media pH 7.4 (Agilent) and cells were incubated at 37°C in a non-CO_2_ incubator for 60 min prior to beginning oxygen consumption readings. The effect on oxygen consumption rate (OCR) was measured 4 times for 2.5 min each in the absence and presence of 1.5 μM oligomycin (to determine coupled respiration), 1.5 μM CCCP (to determine maximal respiration and spare respiratory capacity), and finally 0.5 μM rotenone in combination with 0.5μM antimycin A (to determine proton leak and non-mitochondrial oxygen consumption). Cells were fixed in 4% PFA in PBS after the assay, stained with 10 μM Hoechst for 5 min and imaged on an InCell Analyser (GE Healthcare) to determine cell numbers per well for normalization.

### Immunofluorescent staining

On day 18 of differentiation, cells were fixed in 4% PFA for 30 min. After PBS washes, cells were permeabilized in PBS with 1% Tween (PBST) and 0.1% Triton X-100 for 10 min. Cells were blocked in 5% horse serum in PBST for 1 h and incubated with primary antibodies in PBST overnight at 4°C. Cells were washed in PBST and incubated with secondary antibodies for 1 h at room temperature. Nuclei were stained with 1 μM Hoechst for 5 min prior to imaging. Cells were imaged using an Opera Phenix high content imaging system (Revvity) with a 40x water immersion objective across 20 fields of view and 6 Z stacks. Details of primary and secondary antibodies are provided in Table 2.

**TABLE 2:** List of antibodies used in this study.

Antibody	Species	Dilution used	Supplier
TOM20	Mouse	1:1,000	BD Biosciences (612278)
LC3	Rabbit	1:1,000	MBL (PM026)
LAMP2	Mouse	1:1,000	Santa Cruz (sc-18822)
P62	Rabbit	1:1,000	Proteintech (18420-1-AP)
NDP52	Rabbit	1:1,000	Proteintech (12229-1-AP)
BNIP3	Rabbit	1:200 (immunostaining) 1:1,000 (Western blot)	Abcam (ab109362)
BNIP3L	Rabbit	1:250 (immunostaining) 1:1,000 (Western blot)	Abcam (8,399)
Phospho-BNIP3L (serine 81)	Rabbit	1:1,000	Abcam (ab208190)
HSP60	Chicken	1:5,000	Thermofisher (PA5-143751)
ULK1	Mouse	1:200	Santa Cruz (sc390904)
C9orf72	Rabbit	1:1,000	Atlas (HPA023873)
Tubulin (alpha)	Mouse	1:5,000	Invitrogen (62204)
GAPDH	Mouse	1:2,000	Proteintech (60004-1)
βIII tubulin	Chicken	1:1,000	Merck (AB9354)
MAP2	Rabbit	1:1,000	Abcam (ab32454)
NeuN	Rabbit	1:1,000	Abcam (ab177487)
Anti-mouse Alexa 488 secondary antibody	Goat	1:1,000	A32723
Anti-rabbit Alexa 568 secondary antibody	Donkey	1:1,000	A11037
Anti-chicken Alexa 488 secondary antibody	Goat	1:1,000	A11039
Anti-rabbit HRP secondary antibody	Goat	1:5,000	Dako (P044801-2)
Anti-mouse HRP secondary antibody	Goat	1:10,000	Abcam (ab97040)

### Immunoblotting

Day 18 iNeuron pellets were resuspended in 50 μL lysis buffer consisting of RIPA buffer, protease inhibitor cocktail (Sigma) and phosphatase inhibitor cocktail (Sigma). After 30 min incubating on ice, pellets were centrifuged at 13,000 rpm for 20 min at 4°C. Supernatant was collected and protein content was determined using a Bradford assay as per the manufacturer’s instructions. All samples were denatured at 95°C for 5 min in Laemmli buffer. A 20 μg of protein was loaded on 7.5% or 12% SDS-polyacrylamide gels for resolving, with protein electrophoresis performed using Mini-PROTEAN Tetra Handcast system (Bio-Rad). Proteins were transferred to PVDF membranes (Millipore) at 250 mA for 60 min. Membranes were blocked in 5% milk or BSA in tris buffered saline with Tween20 (TBST). Details of primary and secondary antibodies are provided in Table 2.

### Southern blot protocol for *C9ORF72*-repeat expansions

DNA was extracted from iAstrocyte pellets using the GenElute™ Mammalian Genomic DNA Miniprep Kit (Sigma-Aldrich, G1N350) following manufacturer’s instructions. A 5 μg of DNA was digested using Alul and Ddel restriction enzymes (New England Biolabs) at 37°C for 16 h and electrophoresed in a 1% agarose gel in 1 x TAE. The agarose gel was denatured in 1.5 M NaCl, 0.5 NaOH and neutralized in 3 M NaCl, 0.5 M Tris, pH 7.5. DNA was then transferred to a positively charged nylon membrane (Amersham Hybond N+) by capillary blotting overnight and cross-linked by UV irradiation. A DIG-labeled oligonucleotide probe [5’ (DIG)-(GGGGCC) × 5-(DIG) 3’] was ordered from Thermo Fisher Scientific and denatured at 95°C for 10 min and immediately quenched in ice before use. After 4 h pre-hybridization in DIG Easy Hyb*™* solution (Sigma-Aldrich) at 48°C, the membrane was hybridized with 100 ng/ml of probe and fresh DIG Easy Hyb*™* solution at 48°C overnight in a rotating hybridization oven. The membrane was washed with 2X SSC 0.1% SDS at 48–65°C for 10 min twice, then 0.5 X SSC 0.1% SDS for 15 min, and 0.2 X SSC 0.1% SDS for 15 min at 65°C. The membrane was then washed and blocked using the DIG Wash and Block Buffer Set (Roche) following manufacturer’s instructions, incubated with anti-DIG AP Fab fragments (Roche) at 1:20,000 for 30 min, then washed three times for 10 min in wash buffer. The membrane was then placed in a detection buffer followed by application of CSPD ready-to-use chemiluminescent substrate (Roche), sealed in a plastic sheet, and exposed for 3 h on chemiluminescent film (Amersham, Cat No. 28906837). *C9ORF72*-repeat expansions were quantified from the Southern blot film using an adapted semi-automated protocol (Suh et al., 2015).

### Statistical analysis

Statistical analyses were performed in Prism 10.0 (GraphPad). Normality of data was confirmed with Shapiro-Wilk test. If data were normally distributed, two-tailed unpaired *t*-tests or two-way ANOVAs with Tukey’s multiple comparisons test were performed. A *p* < 0.05 was considered significant. If data were non-normally distributed, Mann-Whitney U tests were performed.

## Results

### C9orf72-HRE reduces mitochondrial membrane potential but not mitochondrial oxygen consumption

**FIGURE 1:**
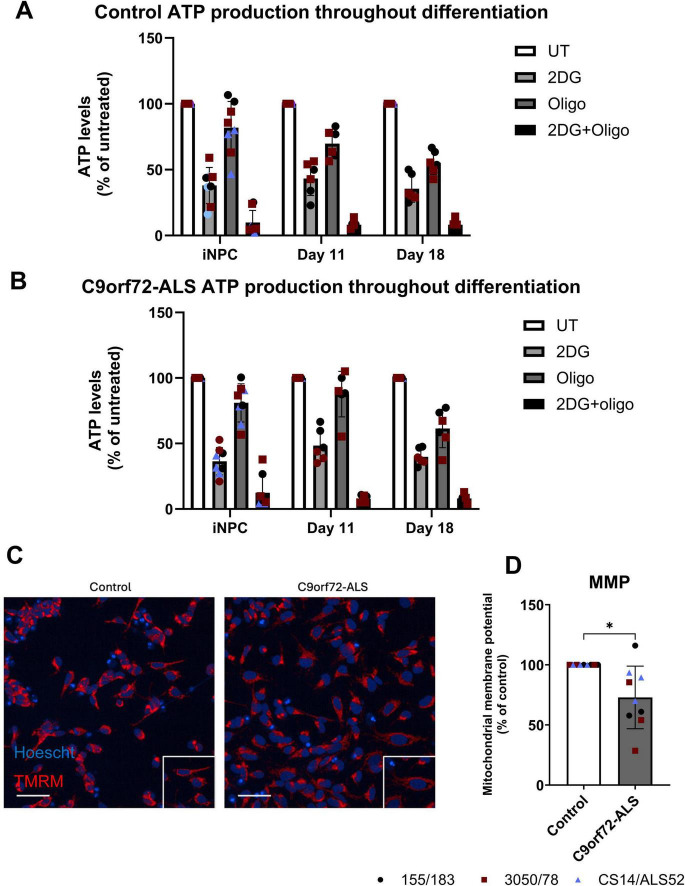
Deficits in mitochondrial function in C9orf72-ALS iNeurons. **(A,B)** ATP production dependent on glycolysis and oxidative phosphorylation throughout differentiation in control **(A)** and C9orf72-ALS **(B)** iNeurons. **(C)** Representative images of control and C9orf72-ALS iNeurons stained with mitochondrial membrane potential marker TMRM. Scale bar = 100 μM. **(D)** Quantification of mitochondrial membrane potential (mean ± SD, Wilcoxon test). All quantification was performed on three different differentiations of 2–3 control and C9orf72-ALS iNeuron lines, each data point represents the mean of three unique differentiations of each control/C9orf72-ALS line, each taken from a mean of approximately 100–500 cells. *p < 0.05.

We first sought to understand mitochondrial function in C9orf72-ALS iNeurons. We have previously shown that this iNeuron differentiation protocol yields neurons that stain positive for pan-neuronal markers β-III tubulin, MAP2 and NeuN, resulting in 83% of neurons at day18 staining positive for β-III tubulin, 75% positive for MAP2 and 52% positive for NeuN (Au et al., 2024; Supplementary Figure 1A), indeed the neurons used in Au et al. (2024) were generated at the same time from the same lines as those used in this study. Furthermore, we assessed the C9orf72 repeat expansion length in these patient iNeurons. C9-ALS lines had repeat lengths of > 1,200 (line 183), 961 (line 52), and 882 (line 78) (Supplementary Figure 1B). To assess the relative contribution of glycolysis and OXPHOS to ATP levels in the iNeurons, we measured ATP levels after treatment with inhibitors of glycolysis (2-deoxyglucose) and OXPHOS (oligomycin). In agreement with previous reports, iNPC’s were found to be heavily glycolytic, with mitochondrial inhibition only reducing ATP levels by ∼19% relative to vehicle treated controls (Schwartzentruber et al., 2020). By the end of differentiation, oligomycin led to larger reductions in ATP levels, down to 55% of UT levels in control iNeurons and 61% in C9orf72 iNeurons (Figures 1A,B). Therefore, these iNeurons have begun the metabolic switch, however, they are still reliant on glycolysis for 50% of their ATP production. No significant difference was observed in ATP levels between control and C9orf72-ALS iNeurons (Supplementary Figure 1).

Next, we stained iNeurons with TMRM and measured fluorescence intensity as an indicator of mitochondrial membrane potential (MMP). We observed a significant decrease in MMP (Figures 1C,D) (*p* = 0.0273). We simultaneously measured mitochondrial morphology using a membrane potential independent dye and in fixed cells using mitochondrial staining of TOM20, however, no differences in mitochondrial area, length, width: length, roundness or density were observed (Supplementary Figure 2). When comparing the distributions of mitochondrial sizes and morphologies we also found no significant differences between control and C9orf72-ALS iNeurone mitochondrial populations (Supplementary Figures 2G–J). Cell size was increased in the C9orf72 iNeurons, however, (Supplementary Figure 1C) (*p* = 0.0056). To further interrogate mitochondrial function, we used the Seahorse Bioanalyser to investigate mitochondrial oxygen consumption under basal and stressed conditions. Due to variability between patient lines, we observed no consistent differences in basal OCR, proton leak, coupled respiration, maximal respiration or spare respiratory capacity (Supplementary Figure 3). Given the iNeurons used in this study still relied on glycolysis for over 50% of ATP production, we also assessed extracellular acidification rate (ECAR) alongside OCR during this assay to provide an indication of glycolytic rate in these iNeurons. We found no significant differences in basal glycolytic rate, glycolytic capacity or glycolytic reserve in C9orf72-ALS iNeurons relative to controls (Supplementary Figure 3).

### C9orf72-HRE leads to reduced basal mitophagy specifically engulfment by the autophagosome

**FIGURE 2:**
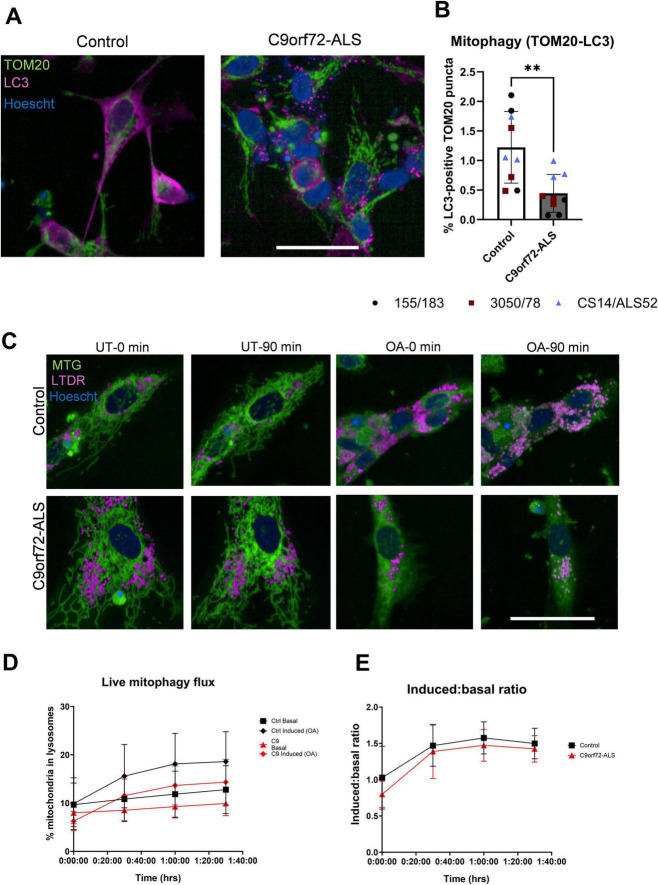
Mitophagy deficit in C9orf72-ALS iNeurons. **(A)** Representative images of control and C9orf72-ALS iNeurons stained with mitochondrial marker TOM20 (green) and autophagosomal marker LC3 (magenta). Scale bar = 100 μM. **(B)** Quantification of percentage of mitochondria co-localizing with autophagosomes (mean ± SD, unpaired *t*-test). Each data point represents the mean of three unique differentiations of each control/C9orf72-ALS line, each taken from a mean of approximately 100–500 cells. **(C)** Representative images of control and C9orf72-ALS iNeurons during mitophagy flux assay in untreated (UT) and induced (OA) conditions. Mitochondria in green, lysosomes in magenta. Scale bar = 100 μM. **(D,E)** Quantification of live mitophagy flux assay and induced: basal ratio. All quantification was performed on 3 different differentiations of 2–3 control and C9orf72-ALS iNeuron lines. **p < 0.01.

Initial assessments of mitophagy were performed by staining for the mitochondrial marker TOM20 and the autophagosomal marker LC3 in fixed iNeurons. A significant decrease in percentage of TOM20-positive mitochondria co-localizing with autophagosomes was observed in C9orf72-ALS iNeurons relative to controls (Figures 2A,B) (*p* = 0.0036). Interestingly, the C9orf72-ALS patient line with the longest repeat length of > 1,200 exhibited the most severe reduction in mitochondria co-localizing with autophagosomes. In contrast, when we investigated mitochondria-lysosome co-localization in iNeurons using live imaging, under basal conditions and after induction with oligomycin-antimycin A, we observed no differences in the number of mitochondria colocalizing with lysosomes (Figures 2C–E).

### C9orf72-HRE disrupts autophagy machinery

**FIGURE 3:**
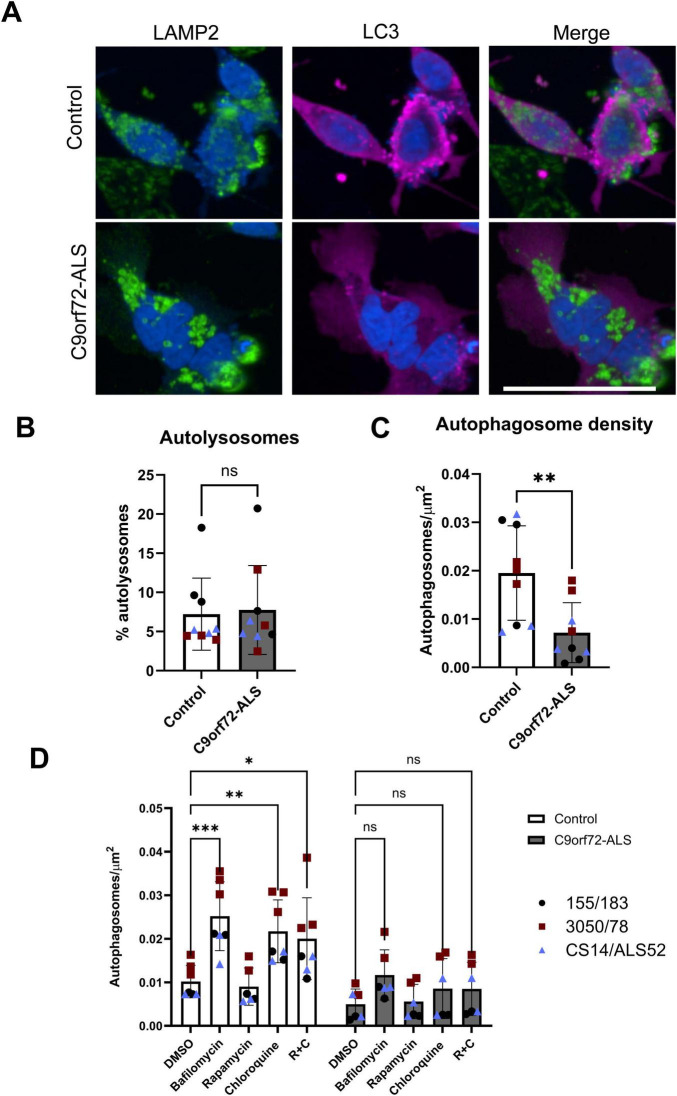
Autophagosome deficit in C9orf72-ALS iNeurons. **(A)** Representative images of control and C9orf72-ALS iNeurons stained with lysosomal marker LAMP2 (green) and autophagosomal marker LC3 (magenta). Scale bar = 100 μM. **(B)** Quantification of percentage of autophagosomes co-localizing with lysosomes (mean ± SD, unpaired *t*-test). **(C)** Quantification of autophagosome density (mean ± SD, unpaired *t*-test). **(D)** Quantification of autophagosome density after treatment with bafilomycin, rapamycin and chloroquine (mean ± SD, 2-way ANOVA with Tukey’s multiple comparisons test). Each data point represents the mean of three unique differentiations of each control/C9orf72-ALS line, each taken from a mean of approximately 100–500 cells. All quantification was performed on 3 different differentiations of 3 control and C9orf72-ALS iNeuron lines. *p < 0.05, **p < 0.01, ***p < 0.0001.

There have been multiple reports indicating a role for the C9orf72 protein and DPR’s on autophagy initiation and autophagosome-lysosome fusion. We stained cells with the lysosomal marker LAMP2 and autophagosome marker LC3. We observed no differences in autophagosome-lysosome co-localization in iNeurons (Figures 3A,B), however, we did observe a reduction in autophagosome density in C9orf72-ALS iNeurons (Figure 3C) (*p* = 0.0056). Once again, the C9orf72-Als line with the most severe reduction is the line with the longest repeat length. We sought to confirm whether autophagy induction was impaired in these iNeurons by manipulating autophagy with well characterized autophagy modulators (Klionsky et al., 2021). iNeurons were treated with bafilomycin A1 or chloroquine, both of which block lysosome degradation of autophagosomes and their contents. Rapamycin was also used as an inhibitor of mTOR, to induce autophagy and autophagosome production, however, in these cells under these conditions rapamycin alone was not sufficient to induce, autophagosome production. There is some debate around the ability of rapamycin to induce autophagy in neurons (Tsvetkov et al., 2010; Rubinsztein and Nixon, 2010). However, treatment with bafilomycin and chloroquine did produce alterations in autophagic flux as expected. Treatments with bafilomycin and chloroquine led to significant increases in autophagosomes in control, but not C9orf72-ALS iNeurons (Figure 3D) (*p* = 0.0004 for bafilomycin treatments and *p* = 0.0095 for chloroquine treatments in Control iNeurons). We observed no differences in lysosomal area or density in C9orf72-ALS iNeurons relative to controls (Supplementary Figures 4A–C).

### PINK1-Parkin and BNIP3/BNIP3L mitophagy pathways are not consistently dysregulated in C9orf72-ALS

**FIGURE 4:**
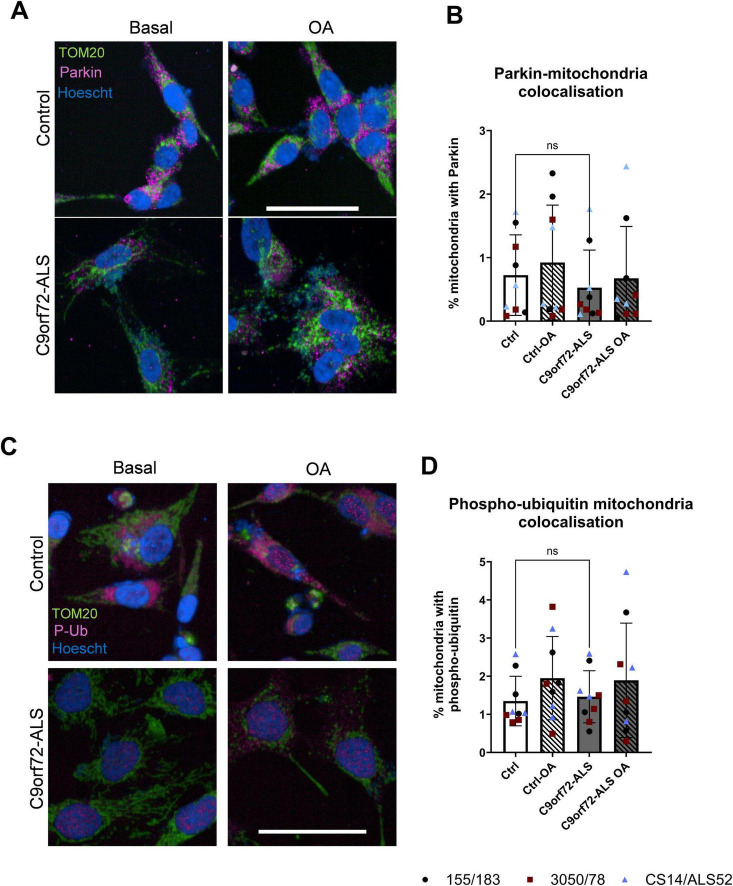
Parkin-dependent mitophagy is unaffected in C9orf72-ALS iNeurons. **(A)** Representative images of control and C9orf72-ALS iNeurons stained with mitochondrial marker TOM20 (green) and Parkin (magenta), under basal conditions and after mitophagy induction with oligomycin/antimycin A (OA) treatment. Scale bar = 100 μM. **(B)** Quantification of percentage of mitochondria staining with Parkin (mean ± SD, unpaired *t*-test). Each data point represents the mean of three unique differentiations of each control/C9orf72-ALS line, each taken from a mean of approximately 100–500 cells. **(C)** Representative images of control and C9orf72-ALS iNeurons stained with mitochondrial marker TOM20 (green) and phospho-ubiquitin (magenta), under basal conditions and after mitophagy induction with oligomycin/antimycin A (OA) treatment. Scale bar = 100 μM. **(D)** Quantification of percentage of mitochondria staining with phospho-ubiquitin (mean ± SD, unpaired *t*-test). Each data point represents the mean of three unique differentiations of each control/C9orf72-ALS line, each taken from a mean of approximately 100–500 cells. All quantification was performed on 3 different differentiations of 3 control and C9orf72-ALS iNeuron lines.

**FIGURE 5:**
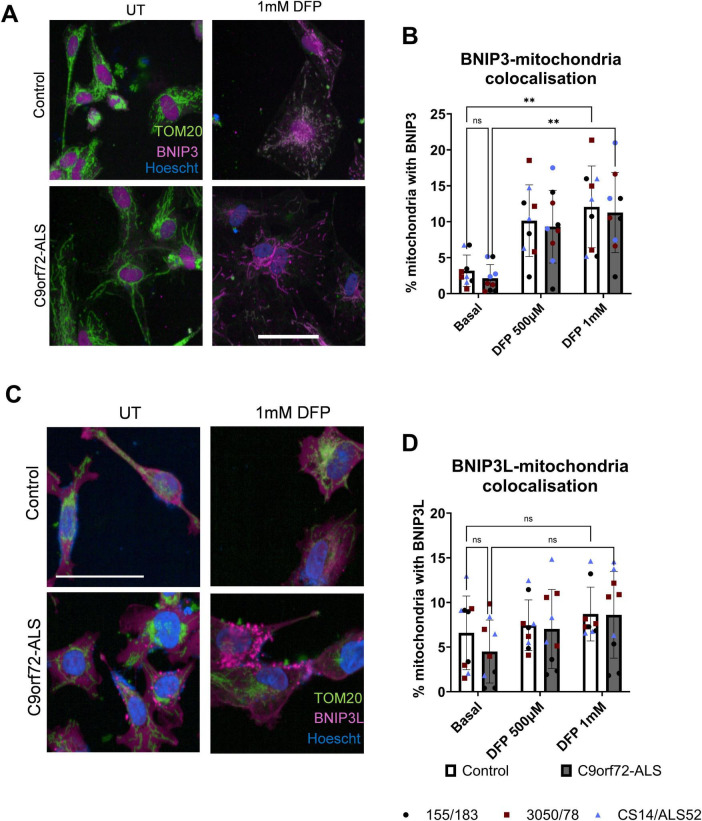
BNIP3-dependent and BNIP3L-dependent mitophagy is unaffected in C9orf72-ALS iNeurons. **(A)** Representative images of control and C9orf72-ALS iNeurons stained with mitochondrial marker TOM20 (green) and BNIP3 (magenta), under basal conditions and after mitophagy induction with deferiprone (DFP) treatment. Scale bar = 100 μM. **(B)** Quantification of percentage of mitochondria staining with BNIP3 (mean ± SD, two-way ANOVA). Each data point represents the mean of three unique differentiations of each control/C9orf72-ALS line, each taken from a mean of approximately 100–500 cells. **(C)** Representative images of control and C9orf72-ALS iNeurons stained with mitochondrial marker TOM20 (green) and BNIP3L (magenta), under basal conditions and after mitophagy induction deferiprone (DFP) treatment. Scale bar = 100 μM. **(D)** Quantification of percentage of mitochondria staining with BNIP3L (mean ± SD, two-way ANOVA). Each data point represents the mean of three unique differentiations of each control/C9orf72-ALS line, each taken from a mean of approximately 100–500 cells. All quantification was performed on 2–3 different differentiations of 3 control and C9orf72-ALS iNeuron lines. **p < 0.01.

We next sought to establish whether common mitophagy initiating pathways upstream of autophagosomal engulfment were disrupted in C9orf72-ALS. Previous studies have shown disruptions to PINK1/Parkin, BNIP3 and BNIP3L-dependent mitophagy in SOD1-ALS, TDP43-ALS, FUS-ALS and sporadic ALS (sALS) neurons (Lagier-Tourenne et al., 2012; Palomo et al., 2018; Rogers et al., 2017; Stribl et al., 2014). We observed no differences in Parkin co-localization with mitochondria in C9orf72-ALS iNeurons relative to controls, although there was variation between the *C9orf72*-ALS patient lines. Downstream of Parkin, we also observed no differences in phospho-ubiquitin accumulation at mitochondria in C9orf72-ALS iNeurons (Figure 4). The recruitment of Sequestosome-Like Receptor proteins NDP52 and P62 were also not disrupted in C9orf72-ALS iNeurons (Supplementary Figure 5). Co-localization of BNIP3, BNIP3L and phospho-BNIP3L with mitochondria basally showed a trend toward being reduced, however, due to large variability between both controls and patients this did not reach statistical significance (Figure 5 and Supplementary Figure 6). After induction with the iron chelator deferiprone (DFP), BNIP3 and BNIP3L localization at the mitochondria was significantly increased in control and C9orf72-ALS iNeurons (Figure 5) (*p* = 0.0017 in control iNeurons, *p* = 0.0011 in C9orf72-ALS iNeurons). Moreover, no changes in expression of BNIP3 and BNIP3L were observed via immunoblot (Supplementary Figures 7, 8). Interestingly, we also observed a significant negative correlation between mitochondrial co-localization and protein expression levels in BNIP3L but not BNIP3 in our iNeuron lines (Supplementary Figures 7E,F), (*p* = 0.0014). Interestingly, as with the mitochondria colocalizing with mitochondria and autophagosome density, the line with the longest C9orf72 repeat expansion showed the lowest BNIP3L levels at mitochondria and highest BNIP3L expression in total.

### ULK1 recruitment is disrupted in C9orf72-ALS iNeurons

**FIGURE 6:**
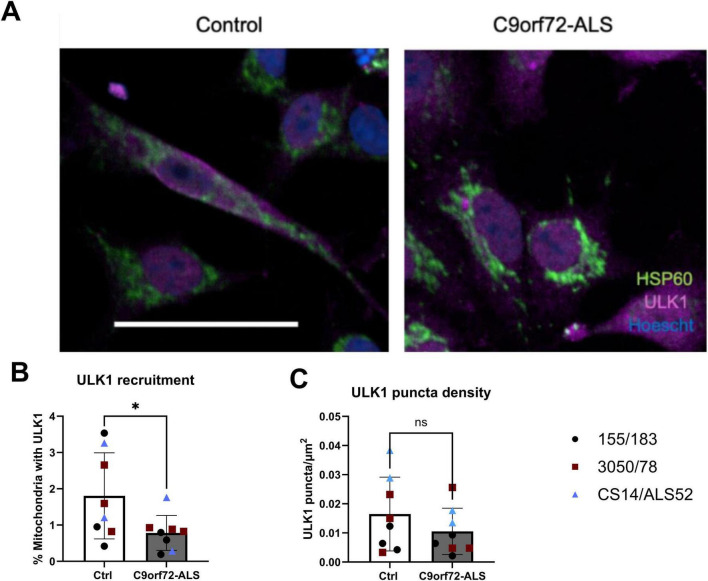
ULK1 recruitment to mitochondria is reduced in C9orf72-ALS iNeurons. **(A)** Representative images of control and C9orf72-ALS iNeurons stained with mitochondrial marker HSP60 (green) and ULK1 (magenta). Scale bar = 100 μM. **(B)** Quantification of percentage of mitochondria staining with ULK1 (mean ± SD, unpaired *t*-test). **(C)** Quantification of ULK1 puncta density (mean ± SD, unpaired *t*-test). Each data point represents the mean of three unique differentiations of each control/C9orf72-ALS line, each taken from a mean of approximately 100–500 cells. All quantification was performed on 2–3 different differentiations of 3 control and C9orf72-ALS iNeuron lines. *p < 0.05.

C9orf72 has previously been shown to mediate the recruitment of the ULK1 complex to the early autophagosome (Sellier et al., 2016; Webster et al., 2016). We investigated co-localization of mitochondria with ULK1 to investigate whether its recruitment to mitochondria prior to mitophagy was disrupted. We observed a significant reduction in ULK1 puncta co-localizing with mitochondria (Figure 6) (*p* = 0.0402). However, we observed no differences in ULK1 puncta density or expression via immunoblot (Figure 6C and Supplementary Figures 9E,F). We also investigated whether increasing ULK1 activity would rescue autophagy deficits caused by this impaired recruitment. Treatment with the AMPK activator A769662 and nilotinib, a drug that has been previously shown to activate ULK1, had no impact on mitophagy or autophagosome production in control or C9orf72 iNeurons. The ULK1 activator BL-918 was the only compound that appeared to have any ability to increase mitophagy in both control and C9orf72-ALS neurons at the highest concentration (increasing mitophagy to 140.7% in control neurons relative to DMSO and increasing mitophagy from 46.3 to 61.7% in C9orf72 neurons relative to DMSO-treated control neurons) although this did not reach statistical significance due to a variable response across the various lines (Supplementary Figure 9). It should be noted here, however, we have not confirmed specific target engagement of these compounds in our model system. Moreover, we show no significant changes in C9orf72 expression in C9orf72-ALS iNeurons (Supplementary Figure 1).

## Discussion

Multiple reports have indicated disrupted autophagy in models of C9orf72-ALS (Beckers et al., 2023; Sellier et al., 2016; Webster et al., 2016). Despite this, the potential impact of C9orf72-HRE mutations on mitophagy remains poorly understood. To address this, we have performed for the first time a comprehensive characterization of mitophagy in C9orf72-ALS iNeurons. Our findings indicate no disruption to mitophagy initiating pathways and suggest that disruption of the autophagy machinery is responsible for reduced basal mitophagy observed in C9orf72-ALS.

We investigated mitochondrial function and morphology in control and C9orf72-ALS iNeurons. Although we observed a significant decrease in MMP, when performing respirometry analysis we observed no significant changes in oxygen consumption (Figure 1D and Supplementary Figure 3). Moreover no changes in mitochondrial morphology were observed (Supplementary Figure 2). Several previous reports have shown reductions in either oxygen consumption or MMP in C9orf72 models (reviewed by Lee et al., 2024). Reductions in basal oxygen consumption and MMP were observed in C9orf72-KO MEF’s, which were accompanied by reductions in Complex I activity and exacerbated by increasing OXPHOS reliance with substitution of glucose for galactose in culture media (Wang et al., 2021). These findings have also been replicated in iPSC-derived motor neurons (Mehta et al., 2021). This apparent discord between MMP and respirometry readings in cells from this study could be due to changes in expression or activity in OXPHOS complexes and may become more pronounced if cells were pushed to greater reliance on mitochondria for ATP production. The identified deficit in MMP is small in the C9orf72-ALS iNeurons and hence over the relatively short differentiation of these cells, may not result in more detrimental consequences. It is also important to note the C9orf72-ALS line with the longest repeat length did not have the most severe MMP deficit, unlike the changes in autophagy and mitophagy we identified. This may indicate different mechanisms are responsible for the mitochondrial functional abnormalities. More detailed analyses of OXPHOS complex function and expression in C9orf72-ALS patient derived models, particularly complex I, may help determine if any of these complexes are uniquely affected by the C9orf72-HRE and are possible targets for therapeutic intervention.

Prior to this study, the impact of C9orf72-ALS on mitophagy was poorly understood. Ectopic expression of the G4C2-HRE in *Drosophila* has previously been shown to reduce mitophagy (Au et al., 2024). However, the cellular mechanisms causing this mitophagy deficit were not explored. There have been multiple reports indicating disruptions to PINK1/Parkin-mitophagy and BNIP3/BNIP3L-dependent mitophagy in TDP43-ALS, SOD1-ALS and sALS in iPSC-neurons, mouse neurons and patient peripheral blood mononuclear cells (PBMC’s) (Araujo et al., 2020; Lagier-Tourenne et al., 2012; Palomo et al., 2018; Rogers et al., 2017; Stribl et al., 2014). Equally, despite conflicting reports, the contribution of the C9orf72-HRE to disrupting autophagy machinery has also been previously described, with reports indicating changes to lysosome function, autophagosome-lysosome fusion and autophagosome production (Beckers et al., 2023; Sellier et al., 2016; Webster et al., 2016; Yang et al., 2016). These previous findings highlight the importance of identifying which specific areas of the mitophagic pathway might be affected to best inform attempts at therapeutic intervention.

PINK1/Parkin-dependent mitophagy is the most well characterized mitophagy pathway, typically triggered when PINK1 stabilizes in the mitochondrial membrane upon mitochondrial injury or depolarization (Jin and Youle, 2012). Subsequently Parkin is recruited, leading to the accumulation of phospho-ubiquitin chains on outer mitochondrial membrane proteins. Previous reports have suggested PINK1/Parkin-dependent mitophagy may be disrupted in ALS, with TDP43-ALS, FUS-ALS and sALS patient derived neurons displaying reduced Parkin expression (Lagier-Tourenne et al., 2012). SOD1 and TDP43 mouse models also display reductions in Parkin expression (Stribl et al., 2014; Rogers et al., 2017). Previous studies have demonstrated differing Parkin dependent mitophagy levels dependent on the metabolic status of the cells (Van Laar et al., 2011; Montava-Garriga et al., 2020; Schwartzentruber et al., 2020). Given the neurons utilized in this study still produced a significant amount of their ATP through glycolysis, this may have influenced the observed phenotypes. Further experiments to determine the impact of oxidative status of the neurons on Parkin-dependent mitophagy would also be useful in these models to determine the impact on this mitophagy pathway.

BNIP3 and BNIP3L have both been identified as mitophagy receptors that act independently of ubiquitination and have been shown to regulate basal mitophagy (Elcocks et al., 2023). Previous studies have identified hypoxia and iron chelation as inducers of BNIP3 and BNIP3L -dependent mitophagy (Allen et al., 2013; Ganley and Simonsen, 2022). We show that under basal conditions, and after induction with deferiprone, there are no differences in BNIP3 or BNIP3L recruitment to mitochondria (Figure 5). Moreover, no differences in expression of either protein were observed under basal conditions (Supplementary Figure 7). However, due to variability in C9orf72-ALS iNeuron lines, further investigations in a larger cohort of patient lines would be useful to accurately establish whether the trending decrease in BNIP3L localization to mitochondria is limited to a small number of patients or widespread in C9orf72-ALS. This will be particularly important as the C9orf72-ALS line which has the most severe reduction in BNIP3L colocalization at the mitochondria is the line with the longest repeat expansion. This patient also had the shortest onset to death time, hence suggesting a more aggressive disease progression. This would be interesting to investigate further in an expanded cohort to understand the mechanisms which correlate with repeat length and disease severity in thinking about targeting these pathways therapeutically. Interestingly, we observed a negative correlation between mitochondrial co-localization and expression levels of BNIP3L but not BNIP3 (Supplementary Figures 7E,F), (*p* = 0.0014 for BNIP3L). Given BNIP3L can interact with other autophagy pathways, this suggests higher levels of BNIP3L under basal conditions may be due to its interactions with other autophagy pathways, something that could warrant further investigation in these iNeurons (Li et al., 2021). We also investigated localization of BNIP3L phosphorylated at serine-81 to mitochondria (Supplementary Figure 6). Although we found no significant difference between control and C9orf72-ALS iNeurons, there were notable reductions in some C9orf72-ALS iNeuron lines relative to their matched controls (Supplementary Figure 6B). Phosphorylation of BNIP3L has been shown to enhance its function in mitophagy (Poole et al., 2021). Again, expanding the cohort size and a more extensive investigation of multiple phosphorylation sites could help identify if reductions in BNIP3L activity, but not expression or mitochondrial co-localization, might impact basal mitophagy in C9orf72-ALS. The drug used to induce BNIP3/BNIP3L-dependent mitophagy in this study, deferiprone, has been assessed in a phase I clinical trial in ALS patients with a phase II/III trial ongoing (Moreau et al., 2018). While the treatment conditions used in this study are a chronic, high dose treatment, we demonstrate increases in autophagy and mitophagy, in agreement with previous work, which may be beneficial in ALS patients if these effects can be achieved with lower doses of deferiprone (Allen et al., 2013).

We identified a deficit in ULK1 recruitment to mitochondria in C9orf72-ALS iNeurons and tried to circumvent this by increasing ULK1 activation by treatment with the compounds nilotinib, BL-918 and A769662. Nilotinib and A769662 have both been shown to activate AMPK leading to ULK1 activation, whilst BL-918 has been shown to activate ULK1 directly (Goransson et al., 2007; Ouyang et al., 2018; Yu et al., 2013). It is important to note, however, we did not measure target engagement of any of these compounds in this study; hence a more detailed study measuring target engagement of small molecules along with genetic manipulation of this pathway is warranted to understand this fully. Interestingly, inhibition of mTOR with rapamycin, a suppressor of ULK1 activity, had no effect in either control or C9orf72-ALS iNeurons. Several previous reports have indicated that inhibition of mTOR with rapamycin or starvation does not induce autophagy in neurons, matching our own data in another neuronal model (Maday and Holzbaur, 2016; Tsvetkov et al., 2010). AMPK activation has recently been shown to enhance clearance of damaged and dysfunctional mitochondria, via Parkin-dependent mitophagy and independently of ULK1, while simultaneously reducing BNIP3L -dependent clearance of functional mitochondria (Longo et al., 2024). These findings warrant a more detailed investigation of AMPK activation as a therapeutic target in C9orf72-ALS iNeuron models.

The morphology and quantity of lysosomes was also measured; however, no significant differences were observed (Supplementary Figure 4). Increases in lysosome size and number have previously been identified in C9orf72-ALS neurons and C9orf72-KO HEK293T cells (Amick et al., 2016; Beckers et al., 2023). Previous reports in C9orf72-ALS neurons do not appear to be driven by C9orf72 expression and may be caused by different levels of DPR’s between their model and the iNeuron model used in this study. Accumulation of autolysosomes has also been reported in *Drosophila* models of C9orf72-ALS expressing DPR’s (Xu et al., 2023). Although no changes in lysosome morphology were observed, changes to lysosomal function were not investigated in this study and cannot be excluded as a contributor to autophagy and mitophagy deficits.

We have identified a deficit in autophagosome production in C9orf72-ALS iNeurons, in agreement with previous reports (Webster et al., 2016; Sellier et al., 2016). Although we hypothesized that reductions in C9orf72 protein expression may be responsible, as a result of the C9orf72-HRE, we did not observe reductions in C9orf72 expression (Supplementary Figure 1). These findings suggest toxic gain-of-function mechanisms associated with the C9orf72-HRE are primarily responsible for driving autophagosomal deficits and may also impact ULK1 recruitment alongside the C9orf72-SMCR8 complex as previously described (Webster et al., 2016; Sellier et al., 2016). Recent studies have indicated that toxic gain-of-function aspects of the C9orf72-HRE, such as DPR production, can contribute to autophagosome and lysosomal dysfunction in C9orf72-ALS. Autophagy deficits have been reported in patient motor neurons but not motor neurons with C9orf72 knockout (Beckers et al., 2023). Poly-PR and poly-GR production can disrupt autophagosome production by enhancing the BCL2-Beclin1 interaction, thereby inhibiting formation of the PIK3C3 complex, downstream of ULK1 in autophagy initiation (Xu et al., 2024). DPR expression in *Drosophila* neurons leads to accumulation of autolysosomes, suggesting that DPR’s interfere with lysosomal degradation (Xu et al., 2023). Taken together, these findings indicate C9orf72 loss and gain of function mechanisms work separately to impair autophagy and highlight the importance of further work in models collectively studying C9orf72 mechanisms, ideally in patient-derived models, to better elucidate any potential corrective therapeutic approaches.

## Data Availability

The original contributions presented in the study are included in the article/Supplementary material, further inquiries can be directed to the corresponding author.

## Supplementary material

Supplementary Figure 1 Neuronal characterization of iNeurons from controls and C9orf72-ALS. **(A)** Quantification of neuron marker expression, previously reported in Au et al. (2024), highlighting differences in expression between different cell lines (mean ± SD). **(B)** Southern blot of patient-derived C9 iNPC lines. Visualization by southern blotting of the C9 repeat expansion in healthy controls and C9-ALS iNPC lines. Bands for the C9-ALS lines correspond to 961 repeats for line 52, 882 repeats for line 78, and over 1,200 repeats for line 183. Bands below 2,000 bp are the result of non-specific binding of the probe to CG-rich regions of the genome. L-DNA Ladder; Ctrl-control plasmid pcDNA3.1/G4C2x45-3xV5, corresponding to a size of 5,527 bp when linear. **(C)** Quantification of ATP levels in control and C9orf72-ALS iNeurons (mean ± SD). **(D)** Quantification of mean cell area in control and C9orf72-ALS iNeurons (mean ± SD). **(E)** Three replicate Western blots for C9orf72 with GAPDH loading control. * represents a non-specific band. **(F)** Quantification of C9orf72 normalized to GAPDH (mean ± SD, unpaired *t*-test).

Supplementary Figure 2 Mitochondrial morphology is unaffected in C9orf72-ALS iNeurons. **(A)** Representative images of control and C9orf72-ALS iNeurons stained with mitochondrial marker TOM20 (green). Scale bar = 100 μM. **(B–F)** Quantification of segmented mitochondria area **(B)**, length **(C)**, roundness **(D)** width:length **(E)** and density **(F)** (mean ± SD, unpaired *t*-test). Each data point represents the mean of three unique differentiation of each control/C9orf72-ALS line, each taken from a mean of approximately 100–500 cells. **(G–J)** Cumulative distribution functions for mitochondrial area **(G)**, length **(H)**, roundness **(I)** and width:length **(J)**. Distributions are for each individual imaged mitochondria from approximately 100–500 cells of each unique differentiation of a control/C9orf72-ALS line, giving a total population of ∼5 million control and ∼5 million C9orf72-ALS mitochondria. All quantification was performed on 3 different differentiations of 3 control and C9orf72-ALS iNeuron lines.

Supplementary Figure 3 C9orf72-ALS iNeurons show no deficits in oxygen consumption. **(A)** Respirometry trace for control (black line) and C9orf72-ALS iNeurons (red line). **(B–G)** Basal respiration **(B)**, proton leak **(C)**, Coupled respiration **(D)**, Maximal respiration **(E)**, Spare respiratory capacity **(F)** and OCR:ECAR ratio **(G)** in control and C9orf72-ALS iNeurons (mean ± SD, unpaired *t*-test). Each data point represents the mean of three unique differentiations of each control/C9orf72-ALS line. All quantification was performed on 3 different differentiations of 3 control and C9orf72-ALS iNeuron lines.

Supplementary Figure 4 Lysosome size and density is unaffected in C9orf72-ALS iNeurons. **(A)** Quantification of lysosome area from LAMP2 staining in control and C9orf72-ALS iNeurons (mean ± SD, unpaired *t*-test). **(B)** Quantification of lysosome area from LysoTracker DeepRed staining during multiple timepoints of mitophagy flux assay in control and C9orf72-ALS iNeurons (mean ± SD). **(C)** Quantification of lysosome density from LAMP2 staining in control and C9orf72-ALS iNeurons (mean ± SD, unpaired *t*-test). Each data point represents the mean of three unique differentiation of each control/C9orf72-ALS line, each taken from a mean of approximately 100–500 cells. All quantification was performed on 3 different differentiations of 3 control and C9orf72-ALS iNeuron lines.

Supplementary Figure 5 P62 and NDP52 co-localization with mitochondria are unaffected in C9orf72-ALS iNeurons. **(A)** Representative images of control and C9orf72-ALS iNeurons stained with mitochondrial marker TOM20 (green) and p62 (magenta). Scale bar = 100 μM. **(B)** Quantification of percentage of mitochondria staining with p62, under basal conditions and after mitophagy induction with oligomycin/antimycin A (OA) (mean ± SD, unpaired *t*-test). **(C)** Representative images of control and C9orf72-ALS iNeurons stained with mitochondrial marker TOM20 (green) and NDP52 (magenta). Scale bar = 100 μM. **(D)** Quantification of percentage of mitochondria staining with NDP52, under basal conditions and after mitophagy induction with oligomycin/antimycin A (OA) (mean ± SD, unpaired *t*-test). Each data point represents the mean of three unique differentiations of each control/C9orf72-ALS line, each taken from a mean of approximately 100–500 cells. All quantification was performed on 3 different differentiations of 3 control and C9orf72-ALS iNeuron lines.

Supplementary Figure 6 Co-localization of phosphorylated-BNIP3L with mitochondria in control and C9orf72-ALS iNeurons. **(A)** Representative images of control and C9orf72-ALS iNeurons stained with mitochondrial marker TOM20 (green) and phospho-BNIP3L (magenta). Scale bar = 100 μM. **(B)** Quantification of percentage of mitochondria staining with pBNIP3LNIX, under basal conditions and after mitophagy induction with Deferiprone (DFP) (mean ± SD, unpaired *t*-test). Each data point represents the mean of three unique differentiations of each control/C9orf72-ALS line, each taken from a mean of approximately 100–500 cells. All quantification was performed on 3 different differentiations of 3 control and C9orf72-ALS iNeuron lines.

Supplementary Figure 7 BNIP3, BNIP3L, and C9orf72 expression are unaffected in C9orf72-ALS iNeurons. **(A)** Representative Western blot for BNIP3L and tubulin. **(B)** Quantification of BNIP3L expression normalized to tubulin. **(C)** Representative Western blot for BNIP3 and tubulin. **(D)** Quantification of BNIP3L expression normalized to tubulin. **(E,F)** Correlations between mitochondrial co-localization and protein expression levels for BNIP3L **(G)** and BNIP3 **(H)** (Pearson’s correlation coefficient). Each data point represents the mean of three unique differentiations of each control/C9orf72-ALS line. All quantification was performed on 3 different differentiations of 3 control and C9orf72-ALS iNeuron lines.

Supplementary Figure 8 Representative full length Western blots for BNIP3L, BNIP3, and tubulin. **(A)** Representative full length Western blot for BNIP3L (left) and tubulin (right) as its corresponding housekeeping gene. **(B)** Representative full length Western blot for BNIP3 (left) and tubulin (right) as its corresponding housekeeping gene.

Supplementary Figure 9 A769662, Nilotinib and BL918 do not rescue mitophagy or autophagosome production in C9orf72-ALS iNeurons. **(A,B)** Quantification of mitophagy **(A)** and autophagosome density **(B)** after A769662 treatment in control and C9orf72-ALS iNeurons (mean ± SD). **(C,D)** Quantification of mitophagy **(A)** and autophagosome density **(B)** after nilotinib and BL-918 treatment in control and C9orf72-ALS iNeurons (mean ± SD). Each data point represents the mean of three unique differentiations of each control/C9orf72-ALS line, each taken from a mean of approximately 100–500 cells. **(E)** Representative Western blot of total ULK1 expression in BL-918 and DMSO-treated iNeurons, with actin as loading control. **(F)** Quantification of expression of ULK1 expression in control and C9orf72-treated iNeurons, following DMSO or BL918 treatment (mean ± SD, unpaired *t*-test). All quantification was performed on 3 different differentiations of 3 control and C9orf72-ALS iNeuron lines. Data is represented as a % of vehicle treated for each line to enable the compound effect to be assessed rather than the basal differences between controls and C9orf72-ALS patient lines.
