# Supplementary figures and images for: C9orf72-ALS mutation drives basal mitophagy impairments in iNeurons
Source: Front Cell Neurosci. 2026 Feb 11;20:1731669. doi: 10.3389/fncel.2026.1731669 (PMC12933941; doi:10.3389/fncel.2026.1731669)

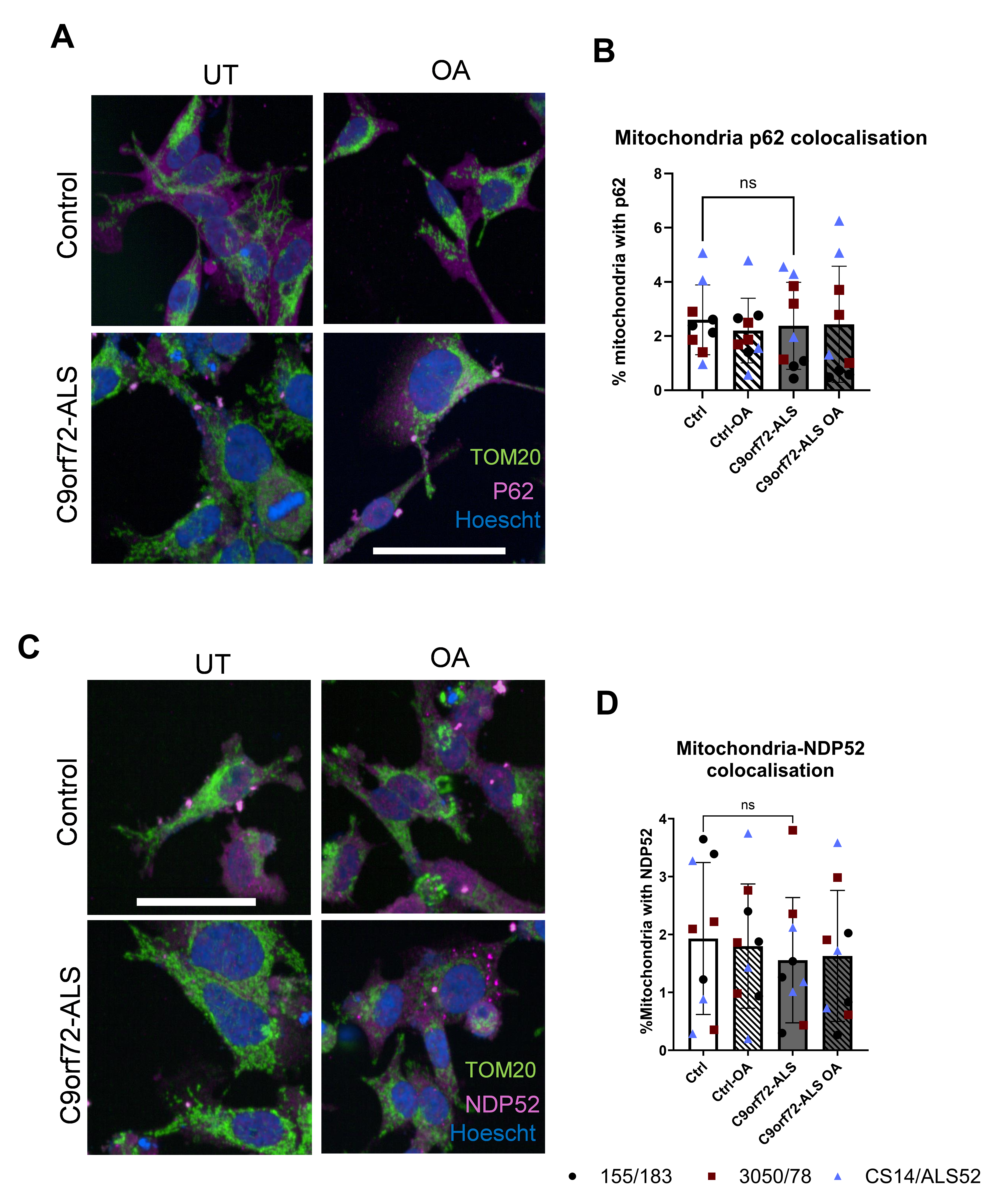

Supplement: Supplementary Figure 5 — P62 and NDP52 co-localization with mitochondria are unaffected in C9orf72-ALS iNeurons. (A) Representative images of control and C9orf72-ALS iNeurons stained with mitochondrial marker TOM20 (green) and p62 (magenta). Scale bar = 100 μM. (B) Quantification of percentage of mitochondria staining with p62, under basal conditions and after mitophagy induction with oligomycin/antimycin A (OA) (mean ± SD, unpaired t-test). (C) Representative images of control and C9orf72-ALS iNeurons stained with mitochondrial marker TOM20 (green) and NDP52 (magenta). Scale bar = 100 μM. (D) Quantification of percentage of mitochondria staining with NDP52, under basal conditions and after mitophagy induction with oligomycin/antimycin A (OA) (mean ± SD, unpaired t-test). Each data point represents the mean of three unique differentiations of each control/C9orf72-ALS line, each taken from a mean of approximately 100–500 cells. All quantification was performed on 3 different differentiations of 3 control and C9orf72-ALS iNeuron lines. [file Image_5.tif]

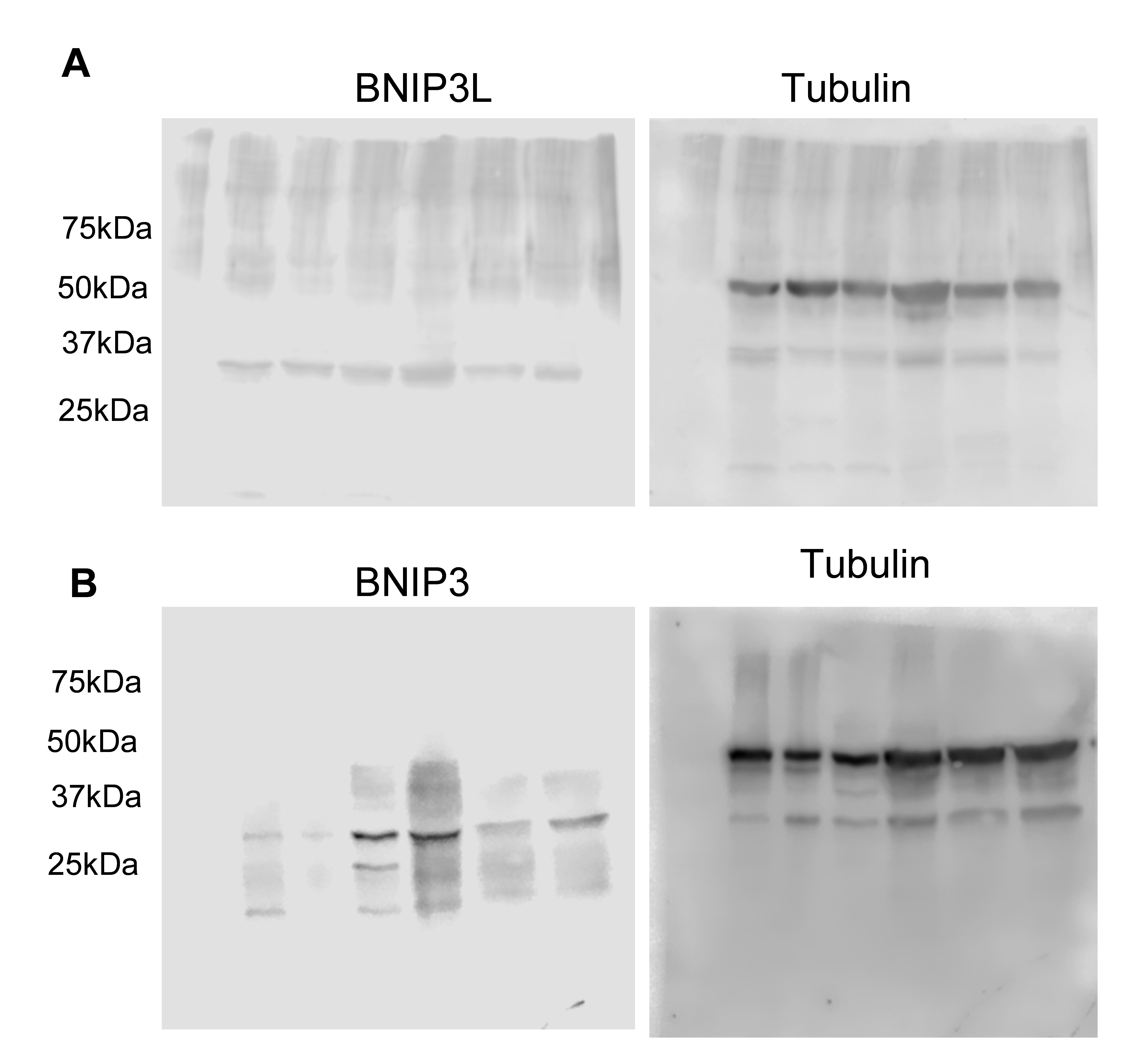

Supplement: Supplementary Figure 8 — Representative full length Western blots for BNIP3L, BNIP3, and tubulin. (A) Representative full length Western blot for BNIP3L (left) and tubulin (right) as its corresponding housekeeping gene. (B) Representative full length Western blot for BNIP3 (left) and tubulin (right) as its corresponding housekeeping gene. [file Image_8.tif]
